# Supplementary material for: Template-controlled on-surface synthesis of a lanthanide supernaphthalocyanine and its open-chain polycyanine counterpart
Source: Nat Commun. 2019 Nov 6;10:5049. doi: 10.1038/s41467-019-13030-7 (PMC6834595; doi:10.1038/s41467-019-13030-7)
Supplement: Supplementary file 1 — Supplementary Information [file 41467_2019_13030_MOESM1_ESM.pdf]

Supplementary Information for

Template-Controlled On-Surface Synthesis of a Lanthanide Supernaphthalocyanine and its Open-Chain Polycyanine Counterpart

Fan *et al.*

Supplementary Discussion.

1) X-Ray Photoelectron Spectra

Supplementary Figure 1 shows the C 1*s* and N 1*s* X-ray photoelectron spectra of the different samples studied by STM. Noteworthy, the XPS data where always recorded exactly at the same samples as were used for the STM data shown in the paper and the Supplementary Discussion. This is possible because the used UHV setup contains both a scanning tunneling microscope and an XPS spectrometer connected with a fast transfer system. The deconvolution of the C 1*s* monolayer spectrum of the ADN monolayer shows a major and a minor component with BEs of 285.0 eV and 286.6 eV, respectively. Due to a higher electronegativity of nitrogen compared to hydrogen and carbon, the C 1*s* signal of the -C≡N group has a higher BE than that of the tetramethyl-tetrahydroanthracene backbone. Therefore, the major and minor components are related to the carbon atoms in tetramethyl-tetrahydroanthracene and carbonitrile (-C≡N) groups of ADN, respectively. The N 1*s* spectrum shows a single peak at 399.6 eV, which is a typical value for nitrogen in a -C≡N···H hydrogen bonding situation. The C 1*s* XP spectrum of Fe-NPc (sample in Figure 2c) shows only one peak located at 284.7 eV. Comparing to the C 1*s* XP spectrum of intact AND, the disappearance of the minor component (related to -C≡N) with a BE of 286.6 eV supports the transformation from the carbonitrile groups in ADN to the pyrrole and aza groups in Fe-NPc. Further evidence is provided by the related changes of the N 1*s* peak, which shifts towards lower BE by 1.7 eV upon formation of Fe-NPc. This result is consistent with the oxidation of iron to Fe(II) and a related gain of negative charge at the N atoms. Note that the BE (397.9 eV) of N 1*s* here is somewhat lower than that reported for the planar iron-phthalocyanine (398.7 eV) on Ag(111) surface. This is explained by the additional gain of electron density from the Ag(111) surface due to the stronger nitrogen-Ag(111) interaction of Fe-NPc than Fe-Pc. The underlying reason for this effect is the tilted adsorption configuration of the lobes (diiminobenzoisoindoline units) of the Fe-NPc molecule, which make the nitrogen atoms pointing down and position them closer to the Ag(111) surface. In addition, a work function change due to the deposition a slight excess of Fe atoms may play a role. The C 1*s* XPS signal of Gd-SNPc (sample in Figure 2e) consists of a single peak located at a BE of 285.1 eV, similar to that for Fe-NPc (284.7 eV) and thus indicating the transformation of the carbonitrile groups to the pyrrole and aza groups. The corresponding N 1*s* peak appears at a BE of 398.2 eV, which is close to that of Fe-NPc (397.9 eV) and indicates a 1.4 eV shift towards lower BE relative to the -C≡N related N 1*s* peak (399.6 eV). For the polycyanine chains, the absence of -C≡N groups is supported by the C 1*s* and N 1*s* spectra of the sample in Figure 3a. Both spectra show single peaks located at BEs of 284.8 eV and 398.1 eV, respectively, shifted toward lower BE compared to the C 1*s* (286.6 eV) and N 1*s* (399.6 eV) peaks related to the -C≡N group.

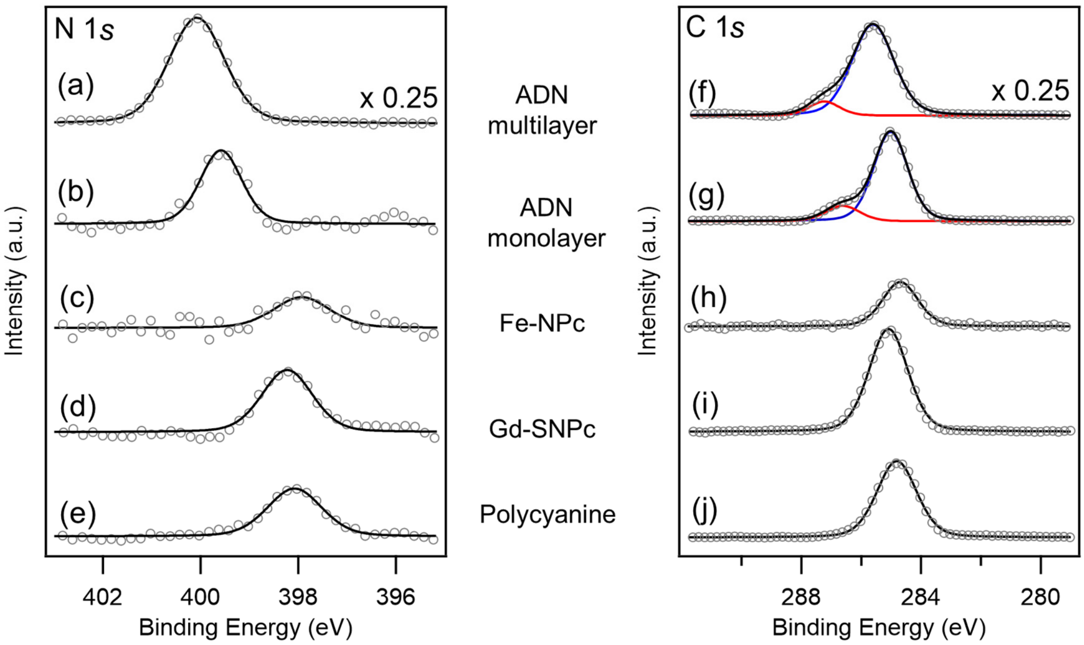

Supplementary Figure 1. N 1*s* and C 1*s* XP spectra of the prepared samples. (a,f) ADN multilayer (5.8 layers), (b,g) ADN monolayer, (c,h) Fe-NPc, (d,i) Gd-SNPc, and (e,j) polycyanine. All spectra were taken at 300 K. The preparation procedure of the corresponding samples can be found in the main text.

2) Structure Confirmation of Fe-NPc by Size Comparison

According to high-resolution AFM images of naphthalocyanine reported previously,[1] the distance (green line, Supplementary Figure 2a) between the centers of two oppositely positioned benzene rings in a naphthalocyanine is measured to be 16.2 ± 0.5 Å as illustrated by Supplementary Figure 2a. The distance (red line, Supplementary Figure 2a) between two adjacent benzene rings is measured to be 2.5 ± 0.5 Å. The corresponding distances of Fe-naphthalocyanine is similar as shown by the Supplementary Figure 2b. Assuming that this distance is close to the distance (red line, Supplementary Figure 2c) between the cyclohexane ring and its adjacent benzene ring, the distance between the centers of two oppositely positioned tetramethyl-cyclohexane units in Fe-NPc is then derived to be 21.2 ± 0.5 Å, as shown by Supplementary Figure 2c. Supplementary Figure 2e-f shows the corresponding experimental distances (20.7 ± 0.5 Å) between the two oppositely positioned tetramethyl-cyclohexane units. This value agrees well with that derived from Supplementary Reference 1. Supplementary Figure 2d illustrates the hypothetical coordination tetramer formed by one Fe atom and four ADN monomers. The chiral vortex arrangement of the four monomer differs completely in appearance from the Fe-NPc with axially arranged four ADN units.

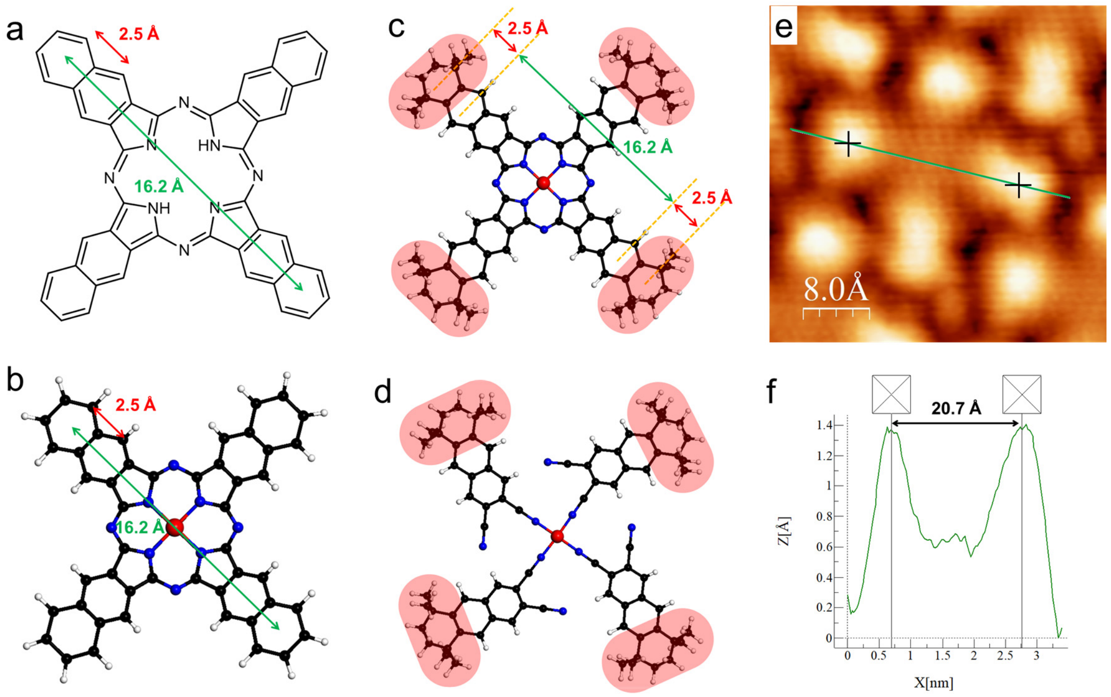

Supplementary Figure 2. Structural analysis of Fe-NPc. (a) Chemical structure of naphthalocyanine labelled with distances derived from its AFM image on NaCl/Cu(111) in ref. 1. The molecular model of (b) Fe-naphthalocyanine with related distances labeled according to the naphthalocyanine, (c) Fe-NPc with distances labeled, and (d) a hypothetical Fe-(AND)$_4$ coordination tetramer. (e) STM image of a single Fe-NPc. Tunneling parameters: U = 1.9 V, I = 0.13 nA. (f) Apparent height profile along the green line in panel (e). The light red ovals in panels (c) and (d) highlight the tetramethyl-cyclohexane moieties, which appear as bright lobes in Fe-NPc and Gd-SNPc. Black spheres represent carbon atoms; blue, nitrogen; white, hydrogen; red, iron.

3) Lateral Distance Uncertainties

The uncertainty of 0.5 Å of the measured distance in Supplementary Figure 2e is derived as show by Supplementary Figure 3 and as discussed in the following. Supplementary Figure 3 shows the apparent height profiles along the green lines overlaid on the STM images. The lobe-to-lobe distances (20.72 Å, 20.45 Å, 20.95 Å, and 20.62 Å) of four cross-shaped species, which are located at different parts of the image, have been measured. Therefore, the average value of these distances is 20.685 Å (≈20.7 Å), and the error bar is derived as 20.95 - 20.685 Å = 0.265 Å (≈0.3 Å). The intrinsic uncertainty from the size of the STM image pixels accounts for another part of the final uncertainty. It is defined as the size of two pixels due to the determination of two peak maxima (2 × 100 Å/512 pixel = 0.4 Å). On this basis, the final maximum uncertainty is estimated to be 0.5 Å. This procedure for the derivation of uncertainties has also been applied to other distance measurements in the main text.

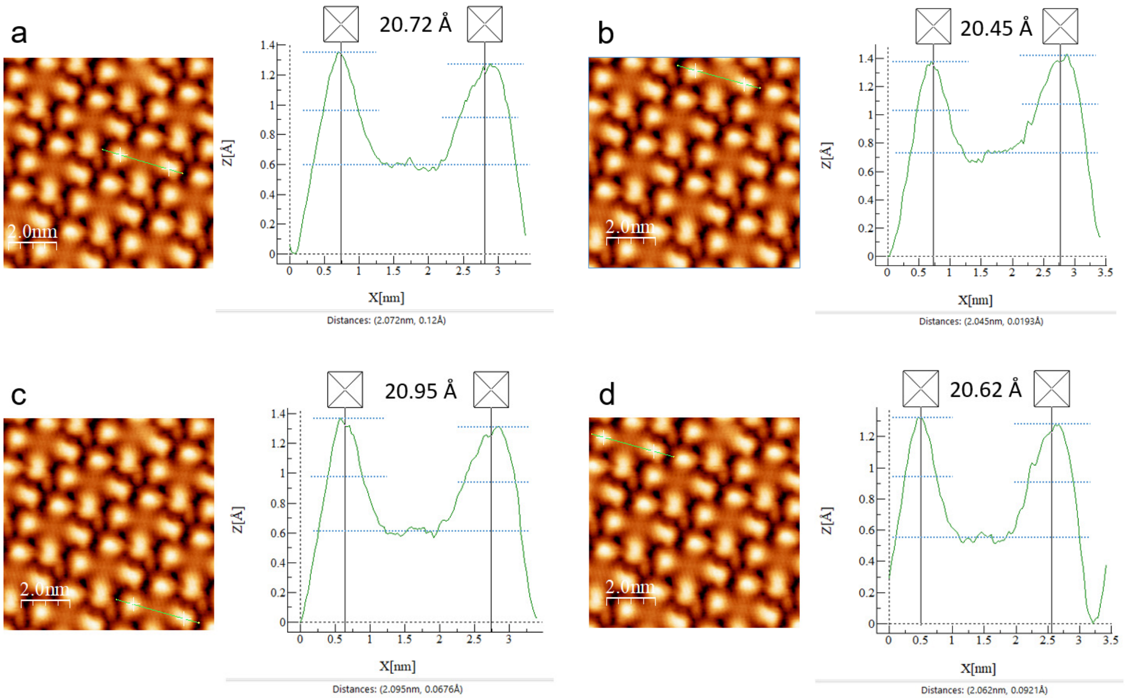

Supplementary Figure 3. Derivation of the uncertainties of the measured lateral distances. (a-d) Apparent height profiles (right part) along the green lines overlaid on different cross-shaped species in the STM images (left part). The three blue dotted lines are given as reference to find the full width at half maximum (FWHM) (the center dotted lines) of the peak. The peak position is defined as the center of the FWHM (black vertical lines).

4) Structure Confirmation of Gd-SNPc by Size Comparison

According to the distance (21.2 ± 0.2 Å, green line, Supplementary Figure 4a) between two opposite tetramethyl-cyclohexane moieties of the Fe-NPc molecule, the distances between the Fe center and the tetramethyl-cyclohexane is derived to be 10.6 ± 0.1 Å. Considering that the N-Gd bond length (2.7 ± 0.5 Å)[2] is about 0.6 Å larger than the N-Fe bond length (2.1 ± 0.5 Å),[3] the distance between Gd and the tetramethyl-cyclohexane moiety in Gd-SNPc is calculated to be 11.2 ± 0.1 Å (Supplementary Figure 4b, red lines). Therefore, the distance between two farthest tetramethyl-cyclohexane moieties in the five-fold symmetric Gd-SNPc molecule is derived to be 21.2 ± 0.1 Å (Supplementary Figure 4b, green line). This agrees well with the experimentally measured distance (21.1 ± 0.7 Å) of two opposite lobes in Gd-SNPc as shown by Supplementary Figure 4c and 4d.

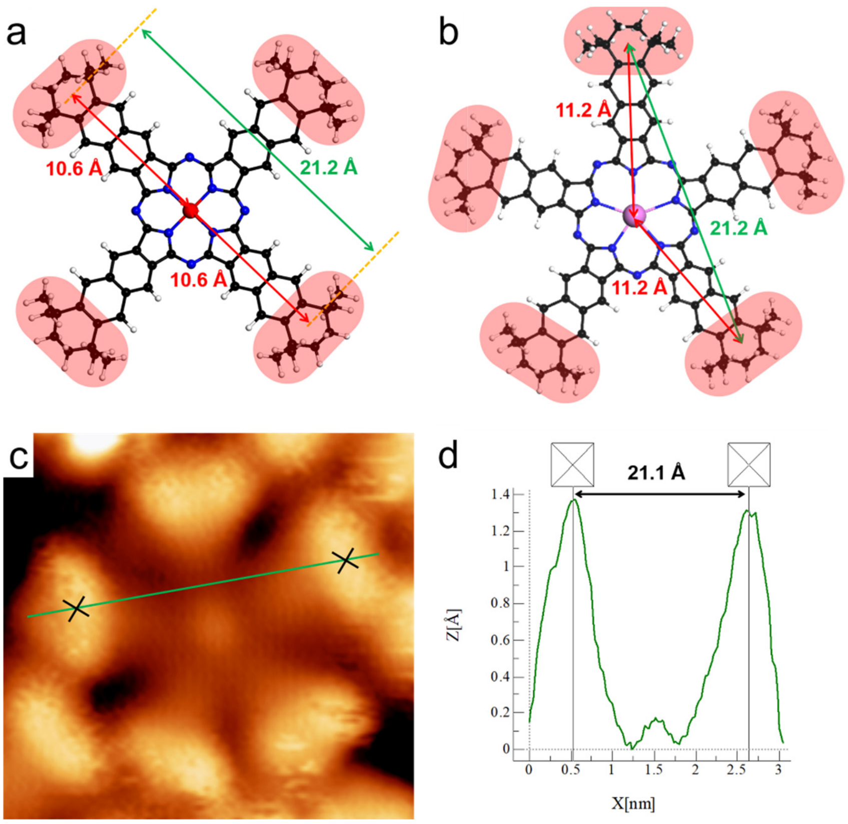

Supplementary Figure 4. Structural analysis of Gd-SNPc. Molecular model of (a) Fe-NPc and (b) SNPc with related distances labeled. The light red ovals in panel (a) and (b) highlight the tetramethyl-cyclohexane moieties, which appear as bright protrusions in Fe-NPc and Gd-SNPc. (c) Magnified STM image of SNPc. Tunneling parameters: U = 1.4 V, I = 0.26 nA. (d) Apparent height profile along the green line in panel (c). Black spheres represent carbon atoms; blue, nitrogen; white, hydrogen; red, iron; pink, gadolinium.

5) Additional High-bias STM Images of Gd-SNPc

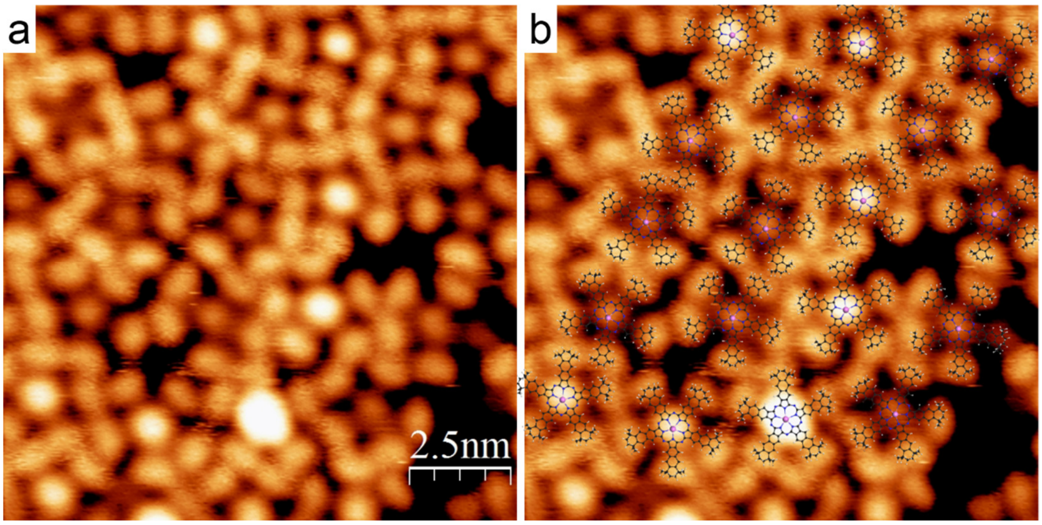

Supplementary Figure 5. STM image of Gd-SNPc assembly recorded with high voltage bias. (a) STM image showing an island of Gd-SNPc with a high tunneling bias. Tunneling parameters: U = 3.3 V, I = 0.10 nA. (b) STM image from panel (a) overlaid with molecular models. Black spheres represent carbon atoms; blue, nitrogen; white, hydrogen; pink, gadolinium.

6) Orientation of a Polycyanine Chain with Respect to the Ag(111) Surface Lattice

The chains extend along the high-symmetry directions of the substrate and have a periodicity of three times the Ag(111) surface lattice constant, *i.e.*, they are commensurate with respect to the substrate. Direct evidence for this is the high-resolution STM image (Supplementary Figure 6) of a single polycyanine chain with the Ag(111) substrate lattice resolved.

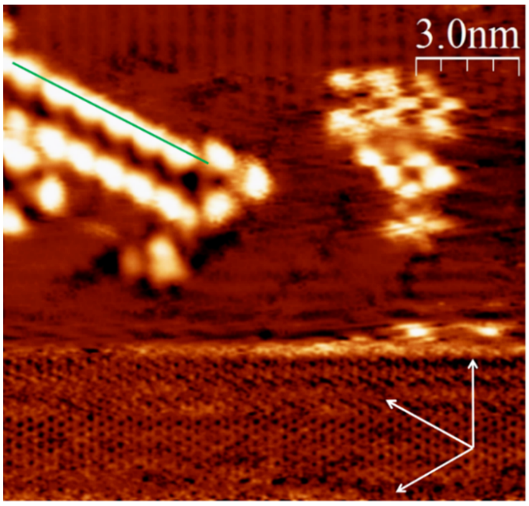

Supplementary Figure 6. STM image of a single polycyanine chain with the Ag(111) surface lattice resolved (in the bottom part). The bottom and top part of the image is recorded with the tunneling parameters U = 0.085 V, I = 0.17 nA and U = 1.3 V, I = 0.1 nA, respectively. The extremely low bias in the bottom part enables the resolving of the Ag(111) surface lattice, allowing the identification of the orientation and the periodicity of the polycyanine chain.

7) Coexistence of Polycyanine Chains and Cyclic Tetramers at Low ADN Coverages

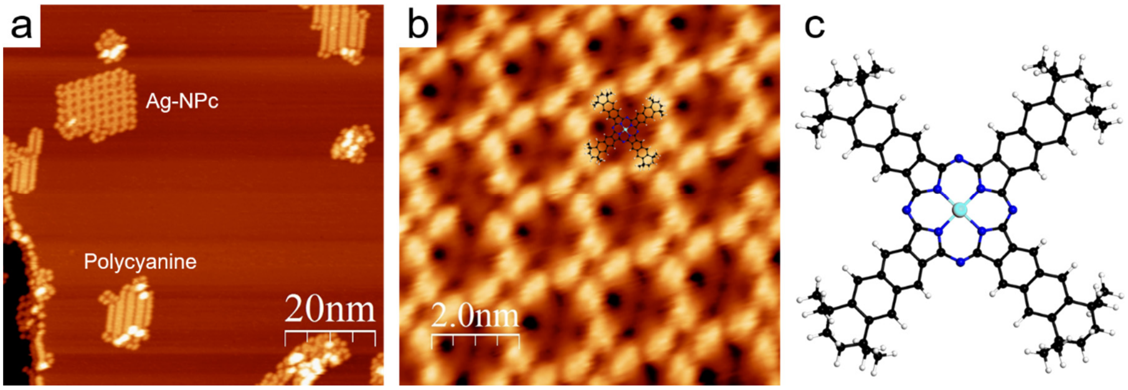

Supplementary Figure 7. STM image showing the coexistence of polycyanine chains and cyclic tetramers. (a) STM image taken after annealing of one monolayer of ADN molecules on Ag(111) to 450 K. The coexistence of polycyanine chains and a cyclic tetramer (tentatively assigned as Ag-NPc) has been observed. (b) Magnified view of the network domain overlaid with the molecular model of hypothetical Ag-NPc. An enlarged image of the molecular structure of the hypothetical Ag-NPc is given in panel (c). Black spheres represent carbon atoms; blue, nitrogen; white, hydrogen; cyan, silver.

8) Conformational Changes Induced by STM Manipulation of the Polycyanine Chains

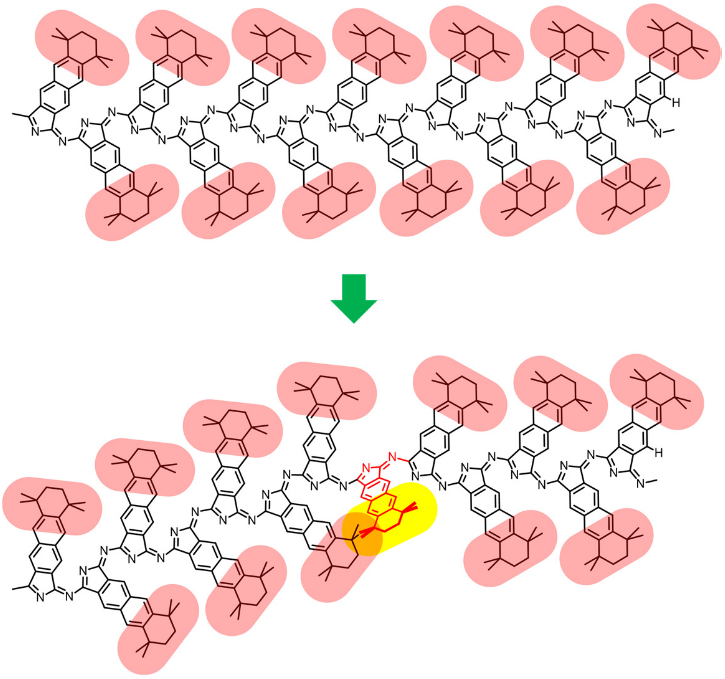

Supplementary Figure 8. Molecular structures of the straight (top) and bent (bottom) polycyanine chains. The bending of a straight polycyanine chain causes the steric repulsion to the benzodiiminoisoindoline units (yellow oval marked) at the corner of a bent chain. This steric repulsion is overcome by the up-lifting of the respective unit.

9) Aromaticity Considerations

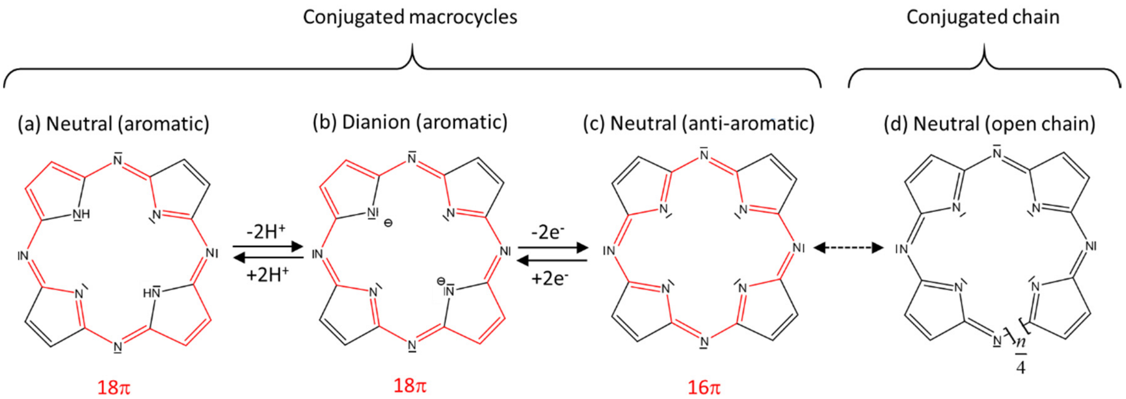

Supplementary Figure 9. Different conjugation pathways in tetraazaporphyrin and its derivatives, compared to the open-chain polymer. (a)-(c) Conjugated tetraazaporphyrin (TAP) macrocycles as models for the phthalocyanine macrocycles. The cyclic systems are stabilized by a Hückel-aromatic 18π electron conjugation path when they contain two NH hydrogens like in the free base (a) or are dianions (b), which occur in metal complexes. If neither condition is fulfilled, an anti-aromatic 16π conjugation path results, as shown in (c). In contrast, conjugation along the open-chain path (d) does not involve aromaticity requirements regarding the electron count. The chain is shown in the quasi-cyclic conformation to illustrate the analogy to the anti-aromatic cyclic system in (c).

10) Bonding Analysis of a Model System with the Periodic Energy Decomposition Analysis (pEDA)

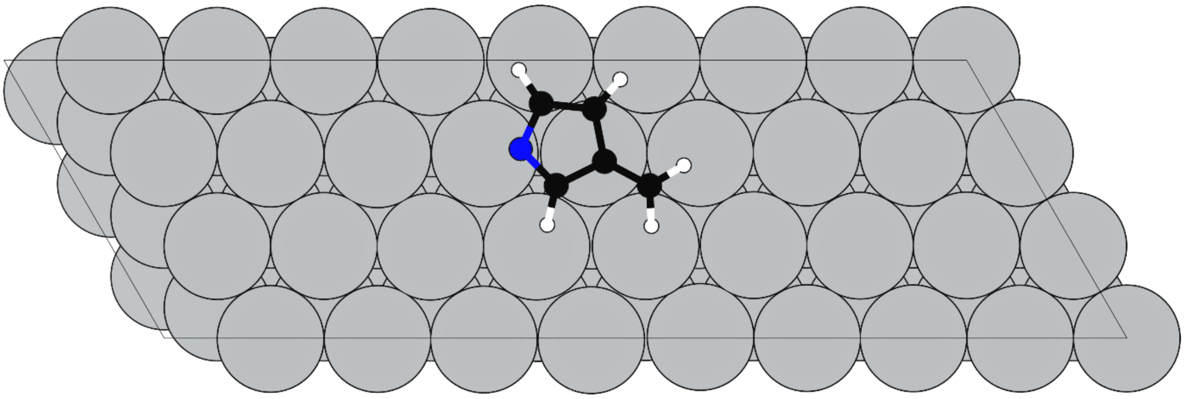

Supplementary Figure 10. Top view of the test system chosen for pEDA. Atoms other than H are frozen in the positions they have in the polycyanine chain.

Supplementary Table 1. pEDA data for the model system (Supplementary Figure 10) with different Monkhorst-Pack type k-meshes (PBE-D3/DZP). Plane wave data for selected energy terms shown for reference. Energies are given in kJ mol^{-1}.

k-mesh	1×1	1×1[a]	3×1	6×2	9×3	6x2[b]
ΔE_{int}	-170	-170	-152	-136	-119	-112
ΔE_{disp}	-89	-89	-89	-89	-89	-89
ΔE_{elec}	-85	-82	-64	-47	-30	-23
ΔE_{Pauli}	483	468	492	489	480	
ΔE_{elstat}	-323	-323	-325	-326	-326	
ΔE_{orb}	-246	-227	-231	-210	-185	

[a] spin-polarized calculation
[b] plane wave calculation with $E_{cut\text{-}off}$ = 400 eV.

Bonding analysis was performed at PBE-D3(BJ)/DZP with different ***k*** space samplings, using pEDA.[4] The pEDA method allows to dissect the interaction energy between two fragments (here: molecule and surface) into well-defined quantities that allow to interpret the bonding in a system in a chemically meaningful way. The interaction energy (ΔE_{int}) is first divided into a dispersion term (ΔE_{disp}) and an electronic term (ΔE_{elec}).

The actual pEDA procedure then decomposes ΔE_{elec} into contributions from Pauli repulsion (ΔE_{Pauli}), electrostatics (ΔE_{elstat}) and orbital interaction (ΔE_{orb}):

$$\Delta E_{elec} = \Delta E_{Pauli} + \Delta E_{elstat} + \Delta E_{orb} \quad (1)$$

This enables a quantitative analysis of the surface-adsorbate bonding.

11) Additional Parameters for the Computed Structure

(a)

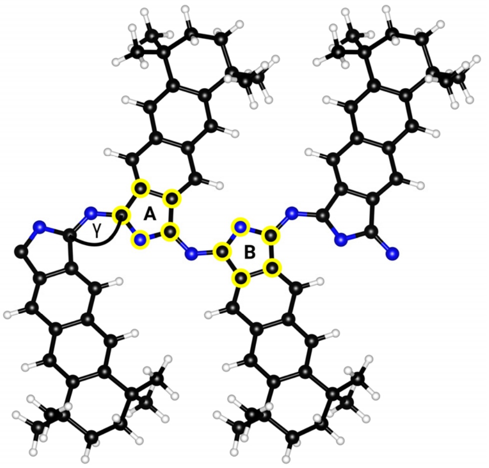

(b)

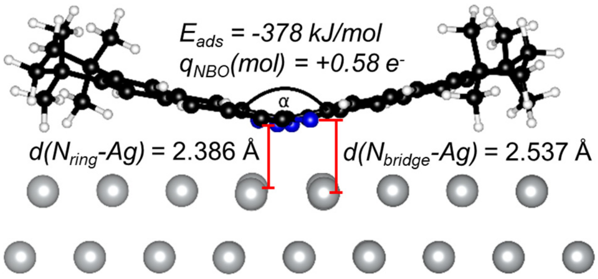

Supplementary Figure 11. Computed structure of polycyanine chains. a) Top view of the chain without the surface. b) Side view of the adsorbed chain with the surface. The charge transfer (q_{NBO}) is calculated with a projection of plane waves[5] onto an atom-centered def2-TZVP basis set.[6] Averaged positions for distance of the N atoms in the ring (N_{ring}) and the aza-bridging N atoms (N_{bridge}) with respect to the non-corrugated surface plane are shown. The adsorption energy term (E_{ads}) is dominated by dispersion interactions (E_{disp} = -412 kJ mol^{-1}) and shows repulsive electronic interactions (E_{elec} = +34 kJ mol^{-1}). The butterfly angle α is defined as an inter-plane angle between the planes spanned by the atoms highlighted in yellow of the two pyrrole rings A and B, respectively.

Supplementary Table 2. Structural parameters of the polycyanine chain free-standing and adsorbed on Ag(111). Definition of angle γ and inter-plane angle α as shown in Supplementary Figure 11, lattice parameter in chain direction.

	Free-standing	Adsorbed
α / °	140.9	155.3
γ / °	130.5	127.2
a / Å[a]	8.67	8.64

[a] the lattice mismatch amounts to 0.4%.

12) Origin of the Unusual Bending Angle of the Polycyanine Chain

The polycyanine chain is not planar, but has an intrinsic bending angle, which is also present in the gas phase calculations. In the following, we will elucidate whether this bending angle is of electronic origin, steric origin, or both. This will be done by using a range of model systems (M1-M8 in Supplementary Figure 12, Supplementary Figures 13 and 14) in which the π-electronic structure and the amount of steric hindrance are systematically varied by substitution or exchange of groups.

Saturation of half of the N atoms in the pyrrole rings (N_{ring}) (M2) reduces bending to 126.62°. The steric repulsion of the N lone pair and –CH in the six-membered "open" ring of M1 is apparently not causing the bending. Instead, it may be of electronic origin. This is indicated by the model system (M3), in which all N atoms in the pyrrole rings (N_{ring}) are saturated. As a result, the bending angle further decreases. Some H-H repulsion prevents an angle of 180°. Removing the H-H repulsion (M4) leads to a perfectly coplanar arrangement. The same is achieved with an all carbon chain (M5). Since isoelectronic substitution does not change the number of π-electrons, another electronic effect must be present. Similarly, saturation of the periphery (M6) only increases the angle to 133.8°. Therefore, the bending effect must be an intrinsic property of the backbone chain.

Saturation of the aza-bridging nitrogen (N_{bridge}) (M7) also results in coplanar arrangement. This is also the case for a larger model system based on M7 (Supplementary Figure 13) containing an additional annulated benzene ring in order to include possible steric repulsion (or, alternatively, CH···N hydrogen bonding) effects between hydrogen on this benzene ring and the lone pair on the N atom in the pyrrole ring (N_{ring}). However, this extended model system also shows coplanar arrangement. Therefore, steric repulsion is ultimately ruled out as the cause of bending.

Instead, the conjugation of the N lone pairs with the π-systems of the chain and the periphery, which emerges as a consequence of the specific arrangement of N and C atoms found in the polycyanine chain, remains as the most probable explanation. Furthermore, adding another imine to the periphery (M8) decreases the angle to 106.9°, the most bent structure in this set.

The larger the electron deficiency is in the π-system, the larger is the stabilization by conjugation. For this reason, saturation with H decreases the bending angle, while adding more electron-withdrawing N atoms increases it. Supplementary Figure 15 shows a magnified version of the frontier orbitals HOCO-2 and HOCO-3 of polycyanine from Figure 5. The conjugation of the N-lone pairs with the π-system of the backbone and the periphery is clearly visible.

Even for a very small model system (M9, Supplementary Figure 14), the bending is found. In the case of this model, we use the dihedral angle θ <(N-C-N-C) to describe the bending since a second ring is not available for the definition of α in the same way as done in the original system. For this model system, we can set up a Walsh diagram (Supplementary Figure 16) showing the orbital shape and energies for different dihedral angles θ. In can be clearly seen that upon increasing the dihedral angle from 0° to the structural minimum of 46° the HOMO-3 representing non-bonding electron pair conjugation is strongly stabilized while the conjugation (orbitals HOMO, HOMO-1, HOMO-4) is decreased by a similar but slightly smaller amount. This intrinsic bending thus stems mostly from the increased conjugation of the non-bonding electron pair at nitrogen.

Noteworthy, to achieve more accuracy of the models in Supplementary Figure 12 and Supplementary Figure 13, we reproduced the GGA results with a range-separated hybrid functional (HSE06) and see the same trends (see the comparisons in Supplementary Figure 12 and 13). Higher level optimization for the molecular model system (MP2) did not show significant deviations in the structure.

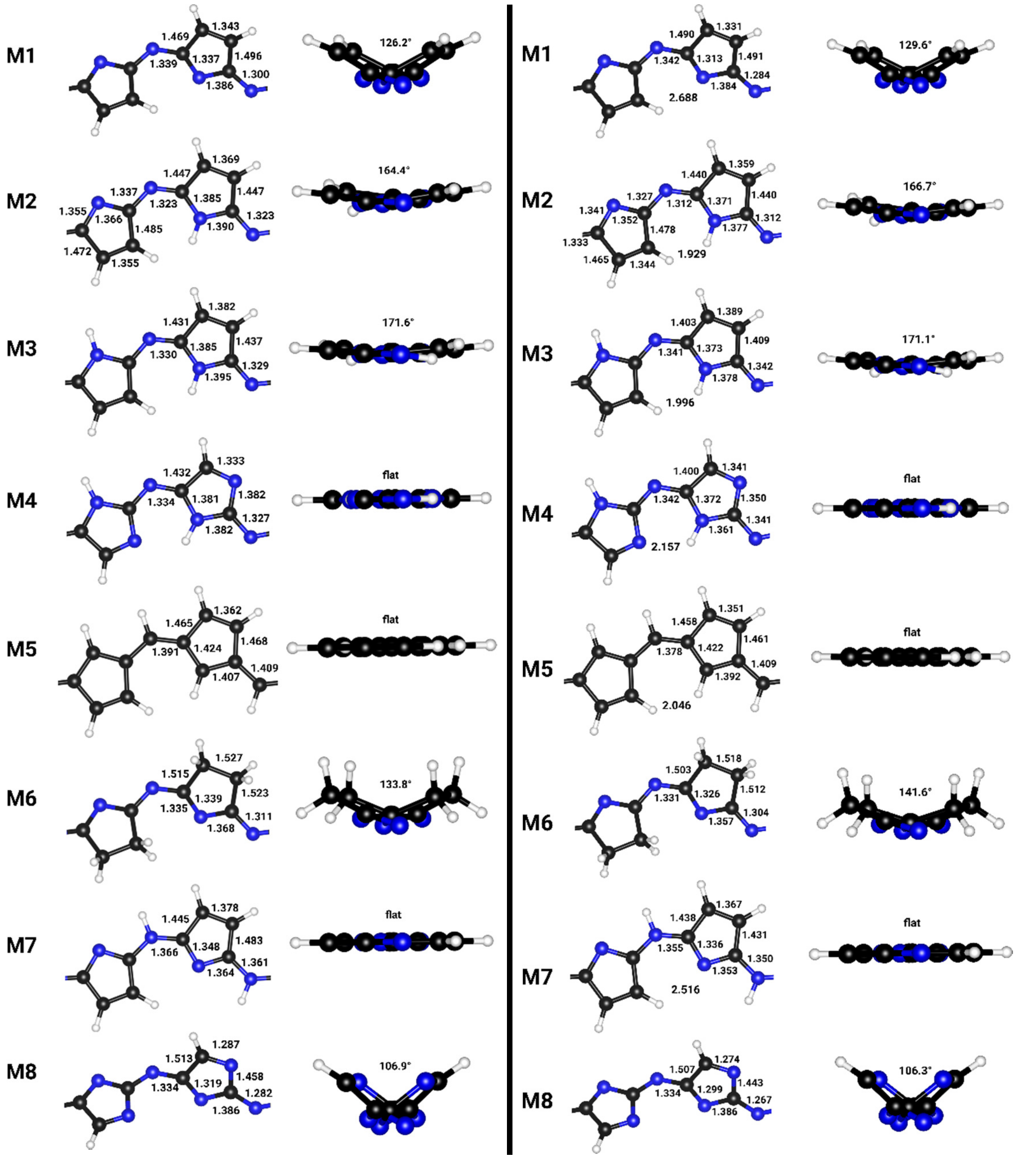

Supplementary Figure 12. Series of model systems for the identification of the origin of the out-of-plane bending (angle defined according to Supplementary Figure 11). (M1) Simplified version of the polycyanine chain. (M2) H saturation of half of the N atoms in the pyrrole rings (N_{ring}). (M3) H saturation of all N_{ring}. (M4) H saturation of all N_{ring} and isoelectronic exchange of a peripheral –CH with N. (M5) Isoelectronic exchange of all N with –CH. (M6) H saturation of the peripheral C=C. (M7) H saturation of all bridging imines (N_{bridge}). (M8) Isoelectronic exchange of a peripheral –CH with N. The calculated models in the left and right columns are obtained by PBE and HSE06 functionals, respectively.

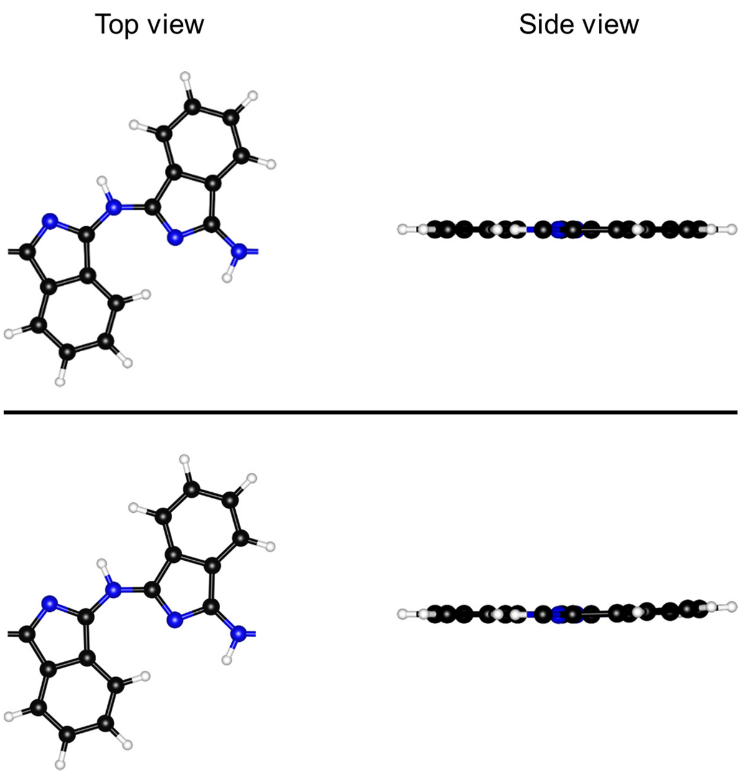

Supplementary Figure 13. A larger model system based on M7, but with an additional annulated benzene ring is planar, excluding steric repulsion (*e.g.* between the H atom at the benzene ring and the lone pair at the N atom in the pyrrole ring) as the cause for the tilted chain. The results at the top and bottom rows are calculated with PBE and HSE06 functionals, respectively.

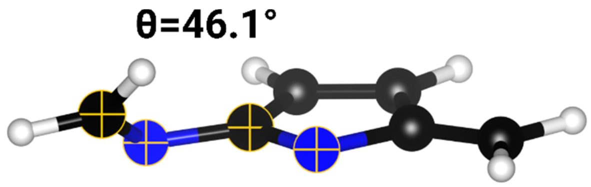

Supplementary Figure 14. A molecular model system (M9) with the same C-N-C-N arrangement as the polycyanine chain shows bending similar to the polymer chain.

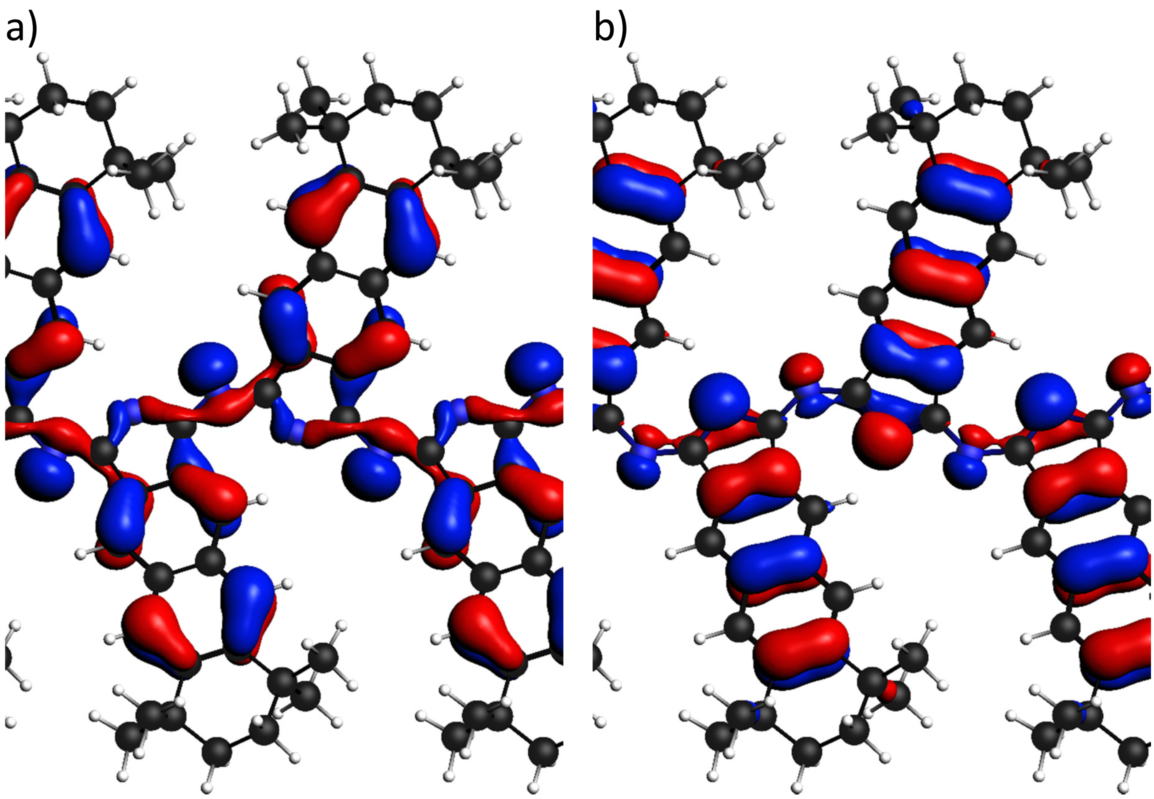

Supplementary Figure 15. Magnified version of the HOCO-2 (**a**) and HOCO-3 (**b**) of the polycyanine from Figure 5 in the main text (isovalue = 0.03). The conjugation of the N-lone pair with the π-system of the backbone (**b**) and the periphery (**a** & **b**) is visible.

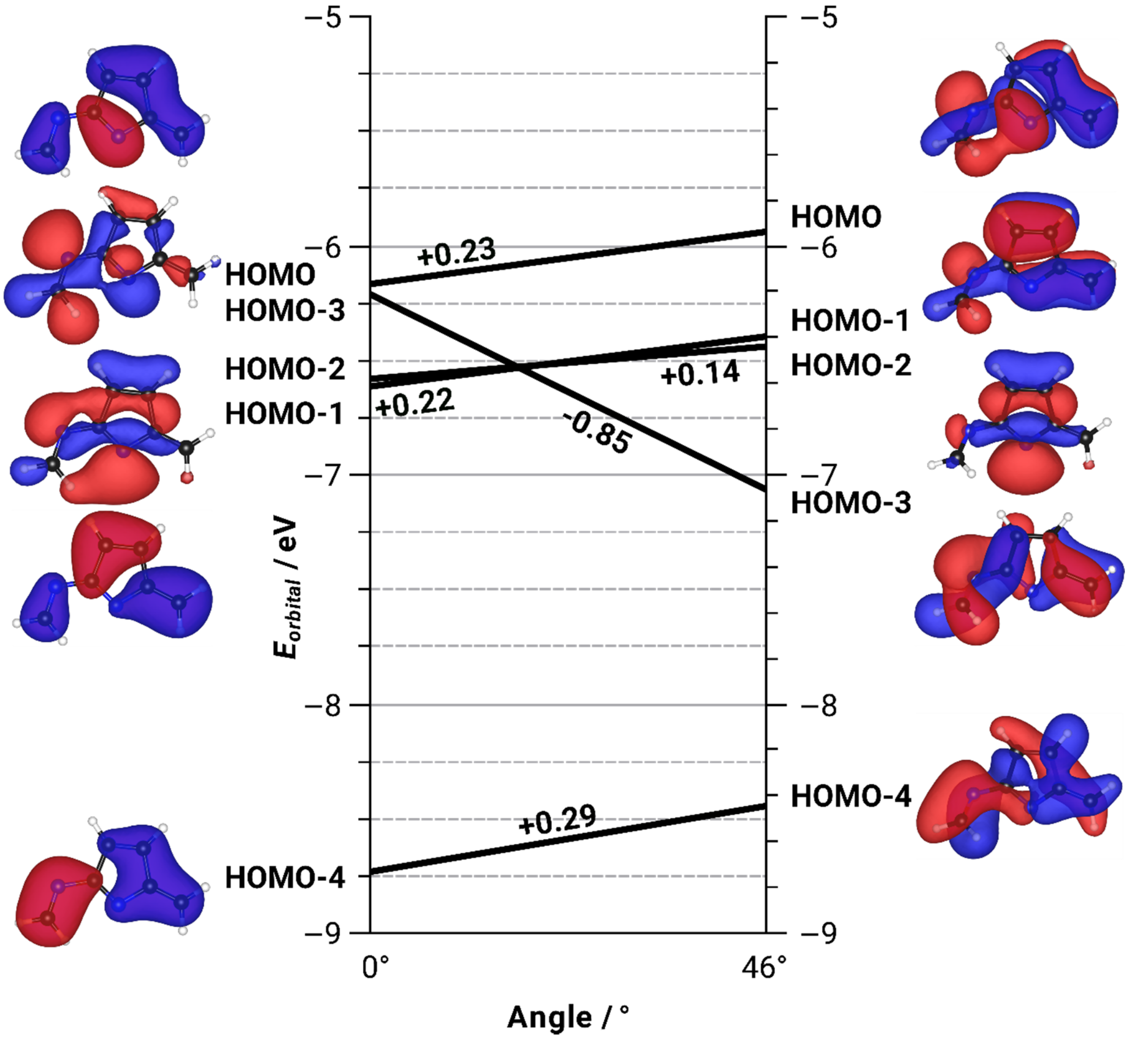

Supplementary Figure 16. Walsh diagram showing the dependence of the canonical Kohn-Sham orbital energies as function of the dihedral angle θ in the model system M9.

13) Electronic Structure of Gd-SNPc and Fe-NPc

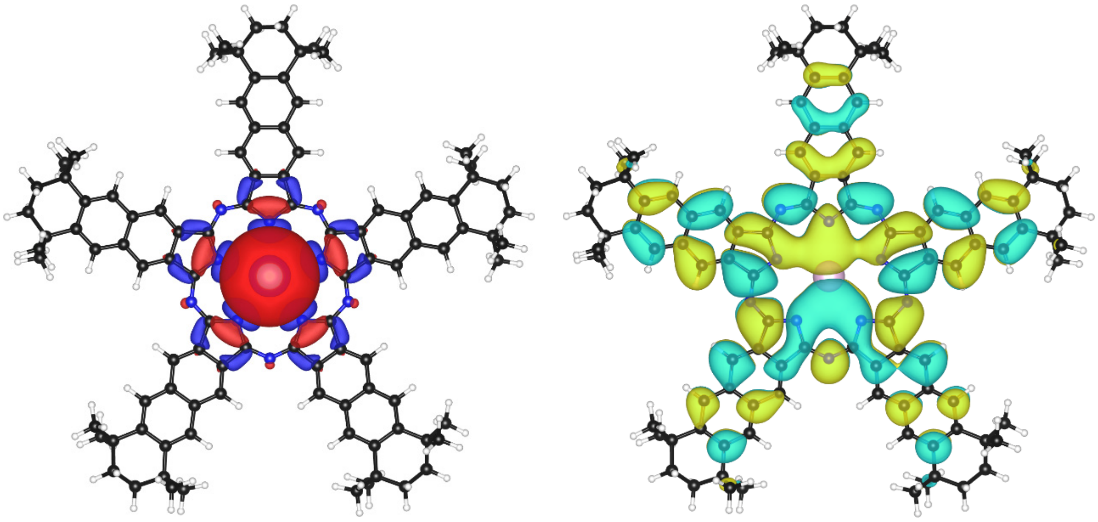

Supplementary Figure 17. Frontier orbitals of Gd-SNPc. Singly-occupied molecular orbital (SOMO, left) and LUMO (right) of the Gd-SNPc as calculated with PBE0. The orbital energy gap amounts to 1.39 eV. Preliminary screening of electronic states with PBE identified eight unpaired electrons as the most favorable state. Seven of the unpaired electrons are located in low-lying 4*f* orbitals, here with α-spin. The eighth unpaired electron, of β-spin, is located in the d_{z^2} SOMO which would favor Gd(II) in the gas phase complex (an all-α configuration could not be converged). These findings indicate a complex electronic structure for Gd-SNPc which is likely not well reproduced with single-determinant methods. The molecule is nevertheless too large for more accurate wavefunction based multireference methods at the current stage. Additionally, on the surface the electronic state is expected to be different.

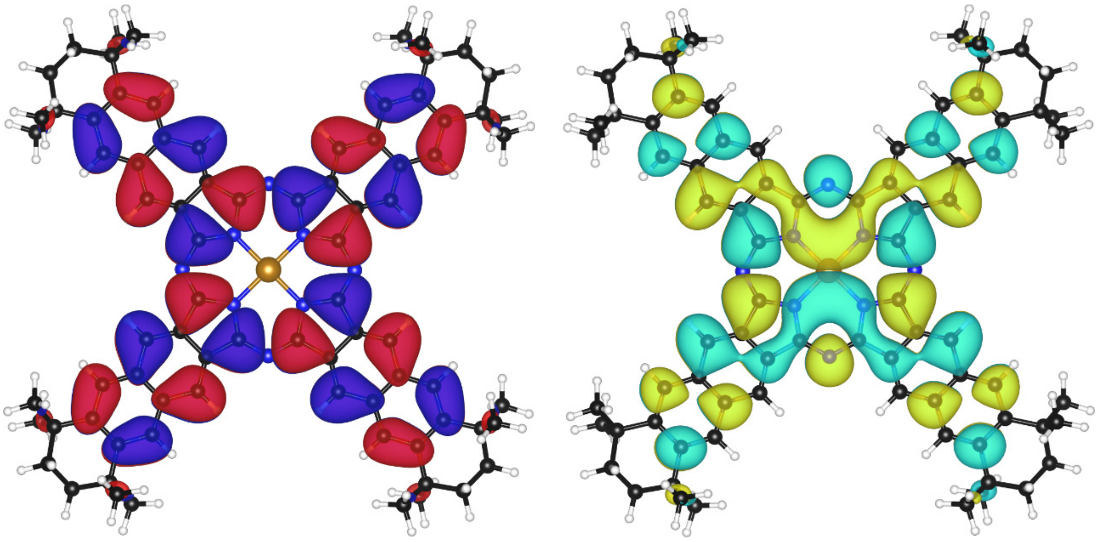

Supplementary Figure 18. Frontier orbitals of Fe-NPc. HOMO (left) and LUMO (right) of the Fe-NPc as calculated with PBE0. The orbital energy gap amounts to 1.71 eV. Of the electronic states investigated, a triplet state was the most favorable in accordance with Fe-PC literature.[7]

14) Optimized Structures in VASP Format

The optimized atomic structures can be found in the NOMAD repositories:
http://dx.doi.org/10.17172/NOMAD/2019.03.18-1
And:
http://dx.doi.org/10.17172/NOMAD/2019.07.01-1

15) ADF/BAND 2017 Input Example

Fragment 1:

```
Accuracy 5
relativistic zora
SCF
Mixing 0.3
Iterations 300
End
Convergence
criterion 1e-6
End
UNITS
  length Angstrom
  angle Degree
END
Atoms C
-0.09450464   -0.64540089   6.00144189
-2.20379164   0.10648111   6.06285689
-0.03599964   0.72955811   6.46990589
-1.34083064   1.18097211   6.51179889
2.11690536   -1.56545189   6.03691489
2.97861936   -2.65075289   6.45969989
-4.35712164   -2.19486689   6.43648289
4.22434036   -0.81019989   5.99716889
End
Atoms N
5.10476136   0.19791011   5.97370089
-1.42231764   -0.98448689   5.73952689
2.89811736   -0.46973389   5.73426389
0.78445436   -1.65376589   5.95038389
End
Atoms H
0.86771436   1.23850211   6.79356789
-1.70970764   2.11766711   6.91097789
-3.45279164   -2.70960989   6.74904089
2.60828736   -3.59945089   6.82687389
End
Lattice
    8.6401377593184261   0.0000000000000000   0.0000000000000000
    4.3200688796592104   7.4825787917669100   0.0000000000000000
End
BasisDefaults
BasisType DZP
Core large
End
XC
LDA LDA
GGA PBE
dispersion Grimme3 BJdamp
END
KSpace
 Grid 3 3
End
skip dos
Dependency basis=1e-8
EndInput
```

Fragment 2:

```
Accuracy 5
```

```
relativistic zora
SCF
Mixing 0.2
Iterations 100
End
Convergence
criterion 1e-6
End
UNITS
  length Angstrom
  angle Degree
END
Atoms Ag
-1.81093864   3.12511111   -3.51784911
1.06910736   3.12511111   -3.51784911
3.94915236   3.12511111   -3.51784911
-4.69098464   -1.86327489   -3.51784911
-1.81093864   -1.86327489   -3.51784911
1.06910736   -1.86327489   -3.51784911
-3.25096164   0.63091711   -3.51784911
-0.37091564   0.63091711   -3.51784911
2.50913036   0.63091711   -3.51784911
-4.69098464   -3.52607089   -1.16630211
-1.81093864   -3.52607089   -1.16630211
1.06910736   -3.52607089   -1.16630211
-3.25096164   -1.03187789   -1.16630211
-0.37091564   -1.03187789   -1.16630211
2.50913036   -1.03187789   -1.16630211
-1.81093864   1.46231511   -1.16630211
1.06910736   1.46231511   -1.16630211
3.94915236   1.46231511   -1.16630211
2.52398236   -2.70599189   1.14001589
-3.20426664   -2.70292489   1.17232689
-0.38249964   -2.70249389   1.16892689
3.95346336   -0.20501789   1.17765489
-1.79878864   -0.22186489   1.19076789
1.10029936   -0.21236889   1.17882189
5.40127736   2.28887911   1.15550789
-0.35609664   2.29087411   1.14698889
2.52441036   2.24692011   1.21120189
-4.63654964   -1.92117789   3.42393889
-1.79183464   -1.88660089   3.62955989
1.07762836   -1.93572189   3.42460589
-3.24477364   0.67610311   3.46137389
-0.31168164   0.66452011   3.45015889
2.53209036   0.60999311   3.68030489
-1.77693964   3.11061711   3.50273689
1.10483336   3.10752911   3.52056289
3.96192936   3.10736811   3.52190589
end
Lattice
    8.6401377593184261    0.0000000000000000    0.0000000000000000
    4.3200688796592104    7.4825787917669100    0.0000000000000000
End
BasisDefaults
BasisType DZP
Core large
End
XC
LDA LDA
GGA PBE
dispersion Grimme3 BJdamp
END
KSpace
 Grid 3 3
End
skip dos
Dependency basis=1e-8
end input
```

PEDA:

```
Accuracy 5
relativistic zora
```

```
SCF
Mixing 0.2
Iterations 200
End
Convergence
criterion 1e-6
End
UNITS
  length Angstrom
  angle Degree
END
fragments f1-mol.runkf
1 1
2 2
3 3
4 4
5 5
6 6
7 7
8 8
9 9
10 10
11 11
12 12
13 13
14 14
15 15
16 16
end
fragments f2-surf.runkf
1 17
2 18
3 19
4 20
5 21
6 22
7 23
8 24
9 25
10 26
11 27
12 28
13 29
14 30
15 31
16 32
17 33
18 34
19 35
20 36
21 37
22 38
23 39
24 40
25 41
26 42
27 43
28 44
29 45
30 46
31 47
32 48
33 49
34 50
35 51
36 52
end
PEDA
Atoms C
-0.09450464   -0.64540089   6.00144189
-2.20379164   0.10648111   6.06285689
-0.03599964   0.72955811   6.46990589
-1.34083064   1.18097211   6.51179889
2.11690536   -1.56545189   6.03691489
2.97861936   -2.65075289   6.45969989
```

```
-4.35712164   -2.19486689   6.43648289
4.22434036   -0.81019989   5.99716889
End
Atoms N
5.10476136   0.19791011   5.97370089
-1.42231764   -0.98448689   5.73952689
2.89811736   -0.46973389   5.73426389
0.78445436   -1.65376589   5.95038389
End
Atoms H
0.86771436   1.23850211   6.79356789
-1.70970764   2.11766711   6.91097789
-3.45279164   -2.70960989   6.74904089
2.60828736   -3.59945089   6.82687389
End
Atoms Ag
-1.81093864   3.12511111   -3.51784911
1.06910736   3.12511111   -3.51784911
3.94915236   3.12511111   -3.51784911
-4.69098464   -1.86327489   -3.51784911
-1.81093864   -1.86327489   -3.51784911
1.06910736   -1.86327489   -3.51784911
-3.25096164   0.63091711   -3.51784911
-0.37091564   0.63091711   -3.51784911
2.50913036   0.63091711   -3.51784911
-4.69098464   -3.52607089   -1.16630211
-1.81093864   -3.52607089   -1.16630211
1.06910736   -3.52607089   -1.16630211
-3.25096164   -1.03187789   -1.16630211
-0.37091564   -1.03187789   -1.16630211
2.50913036   -1.03187789   -1.16630211
-1.81093864   1.46231511   -1.16630211
1.06910736   1.46231511   -1.16630211
3.94915236   1.46231511   -1.16630211
2.52398236   -2.70599189   1.14001589
-3.20426664   -2.70292489   1.17232689
-0.38249964   -2.70249389   1.16892689
3.95346336   -0.20501789   1.17765489
-1.79878864   -0.22186489   1.19076789
1.10029936   -0.21236889   1.17882189
5.40127736   2.28887911   1.15550789
-0.35609664   2.29087411   1.14698889
2.52441036   2.24692011   1.21120189
-4.63654964   -1.92117789   3.42393889
-1.79183464   -1.88660089   3.62955989
1.07762836   -1.93572189   3.42460589
-3.24477364   0.67610311   3.46137389
-0.31168164   0.66452011   3.45015889
2.53209036   0.60999311   3.68030489
-1.77693964   3.11061711   3.50273689
1.10483336   3.10752911   3.52056289
3.96192936   3.10736811   3.52190589
end
Lattice
    8.6401377593184261    0.0000000000000000    0.0000000000000000
    4.3200688796592104    7.4825787917669100    0.0000000000000000
End
BasisDefaults
BasisType DZP
Core large
End
XC
LDA LDA
GGA PBE
dispersion Grimme3 BJdamp
END
KSpace
 Grid 3 3
End
Dependency basis=1e-8
end input
```

16) Synthesis of the ADN Precursor

5,5,8,8-tetramethyl-5,6,7,8-tetrahydroanthracene-2,3-dicarbonitrile (ADN) was prepared in a multistep synthesis *via* 6,7-bis(dibromomethyl)-1,1,4,4-tetramethyltetraline[8] and fumaronitril under reductive coupling conditions:

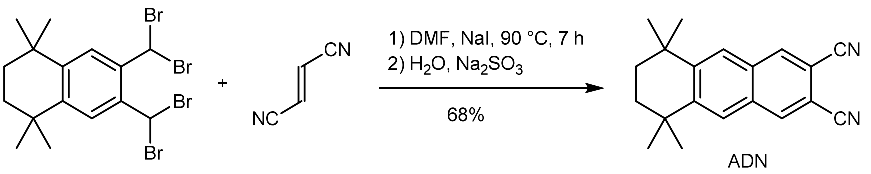

6,7-Bis(dibromomethyl)-1,1,4,4-tetramethyltetraline (10.0 g, 19.0 mmol, 1.0 eq), fumaronitrile (2.63 g, 33.7 mmol, 1.8 eq), and sodium iodide (20.0 g, 134 mmol, 7.1 eq) were dissolved in 20 mL DMF and the solution was stirred at 90 °C for 7 h. After the first 2 h and 4 h, respectively, another two additional portions of fumaronitrile (0.3 g, 3.85 mmol, 0.2 eq each) were added. After 7 h the reaction mixture was cooled down to RT, and 20 mL ice water was added. A brownish precipitate was obtained, which was treated with 50 mL aqueous sodium sulfite solution (10 w%) and isolated by filtration. The product 5,5,8,8-tetramethyl-5,6,7,8-tetrahydroanthracene-2,3-dicarbonitrile (ADN) was purified by column chromatography (silica, toluene) and obtained as brownish powder. Finally, the product was sublimed at 120 °C and 10^{-3} mbar, to obtain ADN as colorless solid.

Yield: 3.75 g, 13.0 mmol, 68%. - ***R_f*** (toluene) = 0.30. - **^{1}H NMR** ($CDCl_3$, 300 MHz): δ = 8.24 (s, 2 H, Ar-C*H*-5, *H*-8), 7.89 (s, 2 H, Ar-C*H*-1, *H*-4), 1.80 (s, 4 H, -CH_2), 1.41 (s, 12 H, -CH_3) ppm. - **^{13}C NMR** ($CDCl_3$, 75 MHz): δ = 150.7, 135.6, 131.6, 126.3, 116.4, 108.9, 35.3, 34.6, 32.5 ppm. - **IR** (ATR, 400-4000 cm^{-1}): $\tilde{\nu}$ = 2961 (s) 2925 (s), 2857 (m), 2349 (w), 2326 (w), 2230 (m), 1623 (w), 1460 (vs), 1366 (m), 1205 (w), 1110 (m), 920 (vs), 542 (m), 528 (w), 467 (vs) cm^{-1}. - **MS** (APCI-HRMS(+)): m/z = 311.1523 $[M+Na]^+$, cal. for $C_{20}H_{20}N_2$+Na_1: 311.1519. - **Elemental analysis** ($C_{20}H_{20}N_2$, M = 288.39 g/mol): fnd. (cal.): C 82.50% (82.30%), H 6.97% (6.99%), N 9.53% (9.71%).

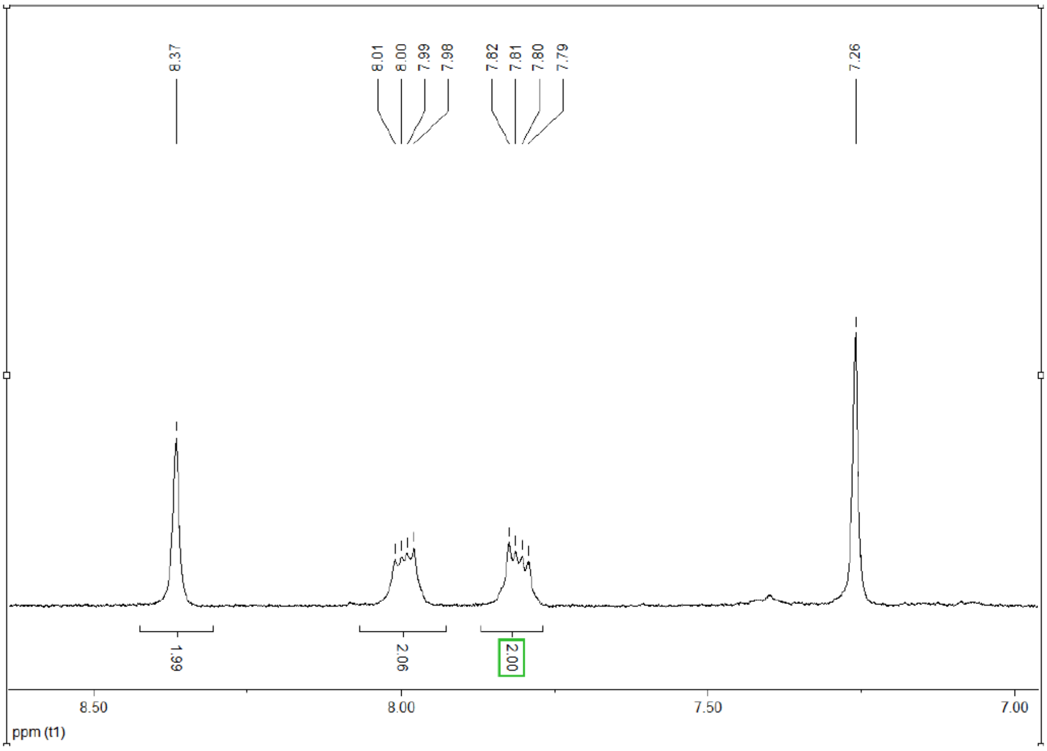

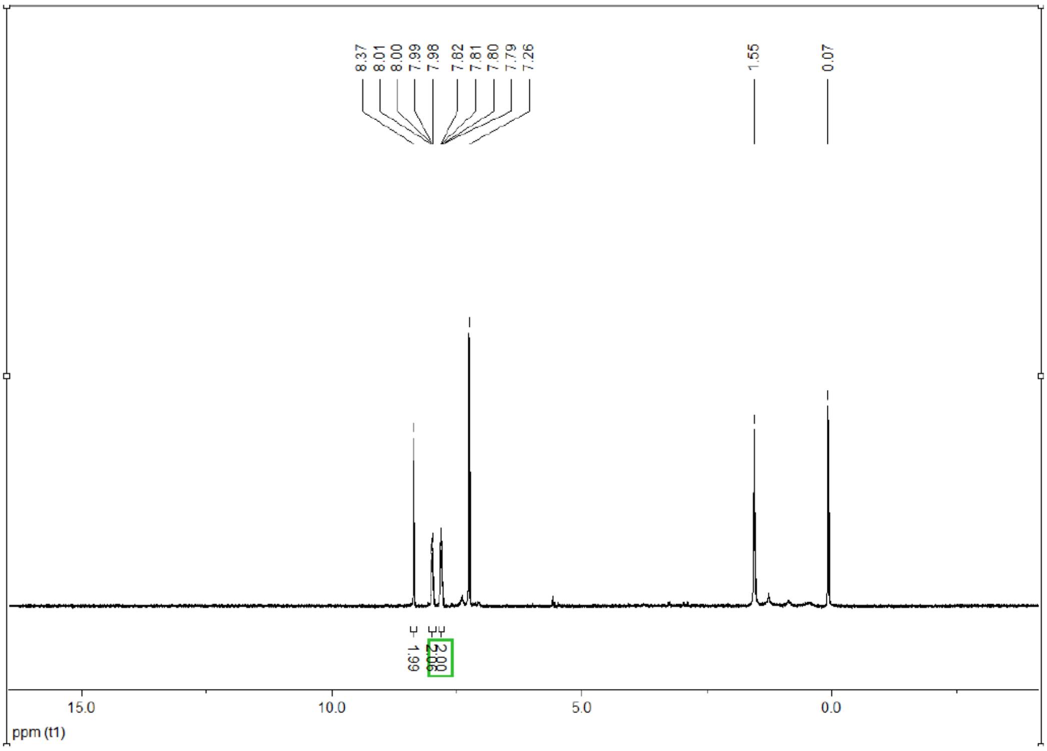

Supplementary Figure 19. 1H NMR of ADN in CDCl3.

Supplementary Methods

1) Details of the DFT Calculations

Density functional theory (DFT) calculations were performed with the Vienna *ab initio* Simulation Package (VASP 5.4.4).[9-12] The generalized gradient approximation-(GGA)-based exchange-correlation functional proposed by Perdew, Burke and Ernzerhof (PBE)[13] was used in conjunction with the projector-augmented wave (PAW) method using the "standard" pseudo potentials for all atoms (version PBE5.4).[14] Dispersion effects were treated with the DFT-D3 scheme using the Becke-Johnson-type damping function.[15, 16] The precision tag was set to accurate, a total energy difference of at most 10^{-5} eV is used for SCF convergence while setting the plane wave energy cutoff to 400 eV. For structure optimization, the force convergence criterion is 10^{-2} eV Å^{-1}. The surface was modelled with the slab-supercell approach using periodic boundary conditions (PBC) and a four-layer 3×9 slab to represent the Ag surface of which the two bottom layers were constrained to the bulk structure. The thickness of the vacuum layer between periodically repeated slabs was larger than 13 Å. The lattice parameter (a = 4.073 Å) was determined computationally using the same input parameters and a 10×10 Γ-centered k-point mesh and 4 atoms per unit cell. This approach delivered accurate results in the past.[17] For the surface calculations, a 6×2 Γ-centered k-point mesh was used. The first order Methfessel-Paxton[18] procedure was used to smear out electronic levels by σ = 0.2 eV to accelerate SCF convergence. This level of smearing corresponds to 2321 K of electronic temperature ($\sigma = k_BT$). Energies are then extrapolated to σ = 0.

The corrugation is calculated with respect to the averaged first layer of the relaxed, pristine surface. STM simulations based on the Tersoff-Hamann approximation using the local density of states were carried out with a bias voltage of -2.4 V. The band structure has been computed without consideration of the surface with the PBE and the HSE06[19] functionals.

Electron density plots, crystal orbitals, and periodic energy decomposition analysis (pEDA) (see below) were calculated with the Amsterdam Density Functional (ADF-BAND 17.107).[20-24] Again, the PBE-D3(BJ) functional was used in conjunction with the atom centered DZP[25-27] basis set. The equivalence of plane wave and atom-centered description of crystal orbitals was confirmed by visual inspection.

Supplementary References.

1. Mohn, F., Gross, L., Moll, N. & Meyer, G. Imaging the charge distribution within a single molecule. *Nat. Nanotechnol.* **7**, 227-231 (2012).

2. Konings, M. S. *et al.* Gadolinium complexation by a new diethylenetriaminepentaacetic acid-amide ligand. Amide oxygen coordination. *Inorg. Chem.* **29**, 1488-1491 (1990).

3. König, E. & Watson, K. J. The Fe-N bond lengths, the "ionic radii" of iron (II), and the crystal field parameters (10Dq) in a high-spin and low-spin [FeII-N6] complex. *Chem. Phys. Lett.* **6**, 457-459 (1970).

4. Raupach, M. & Tonner, R. A periodic energy decomposition analysis method for the investigation of chemical bonding in extended systems. *J. Chem. Phys.* **142**, 194105 (2015).

5. Dunnington, B. D. & Schmidt, J. R. Generalization of natural bond orbital analysis to periodic systems: Applications to solids and surfaces *via* plane-wave density functional theory. *J. Chem. Theory Comput.* **8**, 1902-1911 (2012).

6. Weigend, F. & Ahlrichs, R. Balanced basis sets of split valence, triple zeta valence and quadruple zeta valence quality for H to RN: Design and assessment of accuracy. *Phys. Chem. Chem. Phys.* **7**, 3297-3305 (2005).

7. Brena, B. *et al.* Valence-band electronic structure of iron phthalocyanine: An experimental and theoretical photoelectron spectroscopy study. *J. Chem. Phys.* **134**, 074312 (2011).

8. Mikhalenko, S. A. & Lukyanets, E. A. Phthalocyanines and related compounds. Part 2. Synthesis and certain properties of 2, 3-naphthalocyanines. *J. Gen. Chem. USSR* **61**, 905-910 (1991).

9. Kresse, G. & Hafner, J. *Ab initio* molecular dynamics for liquid metals. *Phys. Rev. B* **47**, 558-561 (1993).

10. Kresse, G. & Furthmüller, J. Efficiency of *ab-initio* total energy calculations for metals and semiconductors using a plane-wave basis set. *Comput. Mater. Sci.* **6**, 15-50 (1996).

11. Kresse, G. & Furthmüller, J. Efficient iterative schemes for *ab initio* total-energy calculations using a plane-wave basis set. *Physic. Rev. B* **54**, 11169-11186 (1996).

12. Kresse, G. & Hafner, J. *Ab initio* molecular dynamics for open-shell transition metals. *Phys. Rev. B* **48**, 13115-13118 (1993).

13. Perdew, J. P., Burke, K. & Ernzerhof, M. Generalized gradient approximation made simple. *Phys. Rev. Lett.* **77**, 3865-3868 (1996).

14. Kresse, G. & Joubert, D. From ultrasoft pseudopotentials to the projector augmented-wave method. *Phys. Rev. B* **59**, 1758-1775 (1999).

15. Grimme, S., Antony, J., Ehrlich, S. & Krieg, H. A consistent and accurate *ab initio* parametrization of density functional dispersion correction (DFT-D) for the 94 elements H-Pu. *J. Chem. Phys.* **132**, 154104 (2010).

16. Grimme, S., Ehrlich, S. & Goerigk, L. Effect of the damping function in dispersion corrected density

functional theory. *J. Comput. Chem.* **32**, 1456-1465 (2011).

17. Rosenow, P., Jakob, P. & Tonner, R. Electron–vibron coupling at metal–organic interfaces from theory and experiment. *J. Phys. Chem. Lett.* **7**, 1422-1427 (2016).

18. Methfessel, M. & Paxton, A. T. High-precision sampling for brillouin-zone integration in metals. *Phys. Rev. B* **40**, 3616-3621 (1989).

19. Heyd, J., Scuseria, G. E. & Ernzerhof, M. Hybrid functionals based on a screened coulomb potential. J. *Chem. Phys.* **118**, 8207 (2003).

20. Velde, G. T. & Baerends, E. J. Precise density-functional method for periodic structures. *Phys. Rev. B* **44**, 7888-7903 (1991).

21. Wiesenekker, G. & Baerends, E. J. Quadratic integration over the three-dimensional brillouin-zone. *J. Phys. Condens. Matter* **3**, 6721-6742 (1991).

22. Franchini, M., Philipsen, P. H. T. & Visscher, L. The becke fuzzy cells integration scheme in the amsterdam density functional program suite. *J. Comput. Chem.* **34**, 1819-1827 (2013).

23. Franchini, M., Philipsen, P. H. T., van Lenthe, E. & Visscher, L. Accurate coulomb potentials for periodic and molecular systems through density fitting. *J. Chem. Theory Comput.* **10**, 1994-2004 (2014).

24. BAND2017, SCM, Theoretical Chemistry, Vrije Universiteit, Amsterdam, The Netherlands, http://www.scm.com.

25. Van Lenthe, E. & Baerends, E. J. Optimized slater-type basis sets for the elements 1-118. *J. Comput. Chem.* **24**, 1142-1156 (2003).

26. Chong, D. P., Van Lenthe, E., Van Gisbergen, S. & Baerends, E. J. Even-tempered slater-type orbitals revisited: From hydrogen to krypton. *J. Comput. Chem.* **25**, 1030-1036 (2004).

27. Chong, D. P. Augmenting basis set for time-dependent density functional theory calculation of excitation energies: Slater-type orbitals for hydrogen to krypton. *Mol. Phys.* **103**, 749-761 (2005).
